# A risk estimation method for depression based on the dysbiosis of intestinal microbiota in Japanese patients

**DOI:** 10.3389/fpsyt.2024.1382175

**Published:** 2024-05-28

**Authors:** Kana Okuma, Kouta Hatayama, Hidetaka Tokuno, Aya Ebara, Ayano Odachi, Hiroaki Masuyama, Naomi Hoshiko, Nobuaki Tanaka

**Affiliations:** ^1^ Symbiosis Solutions Inc., Tokyo, Japan; ^2^ Department of Clinical Biology and Hormonal Regulation, Tohoku University Graduate School of Medicine, Sendai, Japan; ^3^ Hoshiko Holistic Clinic, Tokyo, Japan; ^4^ BESLI Clinic, Tokyo, Japan

**Keywords:** depression, intestinal microbiota, dysbiosis, structural equation modeling, risk estimation

## Abstract

**Introduction:**

Early detection of depression is important for preventing depression-related suicides and reducing the risk of recurrence. This study explored the association between depression and intestinal microbiota and developed a depression risk-estimation method based on this.

**Methods:**

The intestinal microbiota of Japanese patients with depression (33 males and 35 females) and disease-free controls (246 males and 384 females) in their 20’s to 60’s were compared by sex using 16S rRNA gene amplicon sequencing. A depression-risk estimation method was developed using structural equation modeling.

**Results:**

Intestinal bacteria taxa that differed between depression and control groups were identified based on effect size (absolute value greater than 0.2). *Neglecta* was more abundant, while *Coprobacter*, *Butyricimonas*, *Clostridium*_XlVb, and *Romboutsia* were less abundant in the male depression group compared to the male control group. In the female depression group, *Massilimicrobiota*, *Merdimonas*, and *Sellimonas* were more abundant, whereas *Dorea* and *Agathobacter* were less abundant compared to the female control group. Several of the intestinal bacterial taxa that were less abundant in depression were associated with butyrate or hydrogen production. Using these depression-associated intestinal bacteria as indicators, risk-estimation models using structural equation modeling for depression were developed. In the risk-estimation models for males and females, the areas under the receiver operating characteristic curve were 0.72 and 0.70, respectively, indicating that these models can distinguish between individuals with and without depression.

**Conclusions:**

This study provides insights into depression etiology and aids in its early detection and treatment.

## 1 Introduction

The 2019 Global Burden of Disease Study estimated that approximately 280 million people worldwide are affected by depression (depressive disorder) (1, 2). A 2020 survey by Japan’s Ministry of Health, Labor and Welfare reported that the total number of patients with mood disorders, including depression, in Japan was 1,721,000 (3). Therefore, depression is considered a major social problem in Japan.

Presently, depression is the most common mental health disorder worldwide. Depression causes profound distress to affected individuals and their families, impairs social functioning and economic productivity, and leads to premature mortality from suicide and physical illnesses (4). Patients with depression are at high risk of suicidal behavior, and attempts at suicide worldwide (4, 5). Patients with depression may undergo a repeated process of recovery and recurrence. As deaths by suicide occur most frequently during the first depressive episode (4), early identification and appropriate treatment of depression are essential. The risk of recurrence after the first two episodes is substantially lower than that after the third and subsequent episodes (4, 6), and intervention during the first episode may lead to a reduction in the risk of recurrence. International diagnostic criteria, such as the 11^th^ edition of the International Classification of Diseases and the fifth edition of the Diagnostic and Statistical Manual of Mental Disorders, are used to diagnose depression (4). However, these operational diagnostic methods are based on a combination of symptoms. To avoid misdiagnosis, the use of objective testing methods that can aid diagnosis is desirable. Additionally, early detection, diagnosis, and treatment of depression are important because they can prevent depression-related suicides and reduce the risk of recurrence (4). Therefore, there is an urgent need to develop efficient screening methods for depression.

The biological mechanisms underlying the onset of depression are complex and are yet to be fully elucidated (4). Therefore, identifying the molecular mechanisms underlying depression is an urgent requirement. A possible association between the intestinal microbiota and depression has been recently suggested (7–10). A study conducted in European participants using the Rotterdam Study cohort and the Amsterdam HELIUS cohort reported that 13 bacterial taxa (*Eggerthella*, *Subdoligranulum*, *Coprococcus*, *Sellimonas*, *Lachnoclostridium*, *Hungatella*, *Eubacterium ventriosum* group, *Ruminococcus gauvreauii* group, *Lachnospiraceae*, and four taxa belonging to *Ruminococcaceae*) were associated with depressive symptoms (10). These bacteria are known to be involved in the synthesis of glutamate, butyrate, serotonin, and gamma amino butyric acid (GABA), which are key neurotransmitters in depression. A study on a Japanese population suggested that *Alistipes*, *Blautia*, *Coprococcus*, *Dorea*, *Faecalibacterium*, and *Oscillibacter*, which are associated with butyrate production, are associated with depression, and that the intestinal microbiota may influence the depressed state of the host via the butyrate-producing process (9). In contrast, the study reported that no statistically significant relationship was observed regarding the association of depression with interleukin-6 (IL-6) levels and daily intake of soluble fiber. This Japanese study was performed based on the hypothesis that the translocation of inflammation-induced intestinal microbiota into the intestinal epithelium accelerates the release of immune mediators, such as IL-6, which leads to increased levels of circulating stress hormones or impacts the central nervous system, ultimately exacerbating symptoms of depression; however, no results were found to support this hypothesis. Moreover, consensus on the taxa of the intestinal bacteria associated with depression is lacking (7), and more studies are necessary to clarify this association.

The prevalence of depression differs based on sex, with females twice as likely as males to develop depression (11). In addition, sex differences are also present in the intestinal microbiota. A study in a Japanese population showed that many of the taxa of intestinal bacteria that were potentially associated with disease differed between the sexes (12). Considering the sex differences in the prevalence of depression and intestinal microbiota composition, studies investigating the association between depression and intestinal microbiota should be stratified according to sex. However, many of the previous studies were conducted with mixed populations consisting of both males and females.

Estimating disease risk based on intestinal microbiota composition using structural equation modeling (SEM) has been previously reported. Risk-estimation methods have been developed for atopic dermatitis and mild cognitive impairment based on their association with intestinal microbiota (13, 14). Therefore, this method could provide a risk-estimation model for depression which could subsequently be a promising predictive method for depression if indeed the risk of depression is associated with intestinal microbiota. This predictive method can be incorporated into the testing of intestinal microbiota using stool samples. The intestinal microbiota test is not invasive, and sample collection can be performed at home, which reduces the psychological burden on the participant. If this is used not only for self-care, but also in healthcare settings and during regular health checkups, it could facilitate the early detection of depression. Therefore, this study investigated the intestinal bacterial taxa associated with depression and constructed a method to estimate the risk of depression based on the composition of the intestinal microbiota. Owing to the known sex differences observed in depression prevalence (11) and in the composition of intestinal microbiota, these analyses were performed based on sex.

## 2 Materials and methods

### 2.1 Study population and background information

Data from 2,487 study participants were collected between April 2020 and October 2022 by Symbiosis Solutions Inc. (Tokyo, Japan). All participants provided written informed consent before participating in the study. The study was conducted in accordance with the guidelines of the Declaration of Helsinki and was approved by the Institutional Review Board of Shiba Palace Clinic (Tokyo, Japan) (approval number and date: 144131_rn-27593, January 9, 2020; 145968_rn-29327, November 12, 2020).

The participants were tested using the Center for Epidemiologic Studies Depression Scale (CES-D) (15). The CES-D is a 20-item self-report depression symptom scale. The background information (including age, sex, height, weight, pregnancy and lactation status, antibiotic and enema use, and disease status) of the participants was collected through self-report via a questionnaire that was administered at the time of stool sample collection.

Some participants reported having been diagnosed with depression by a physician.

**Figure 1:**
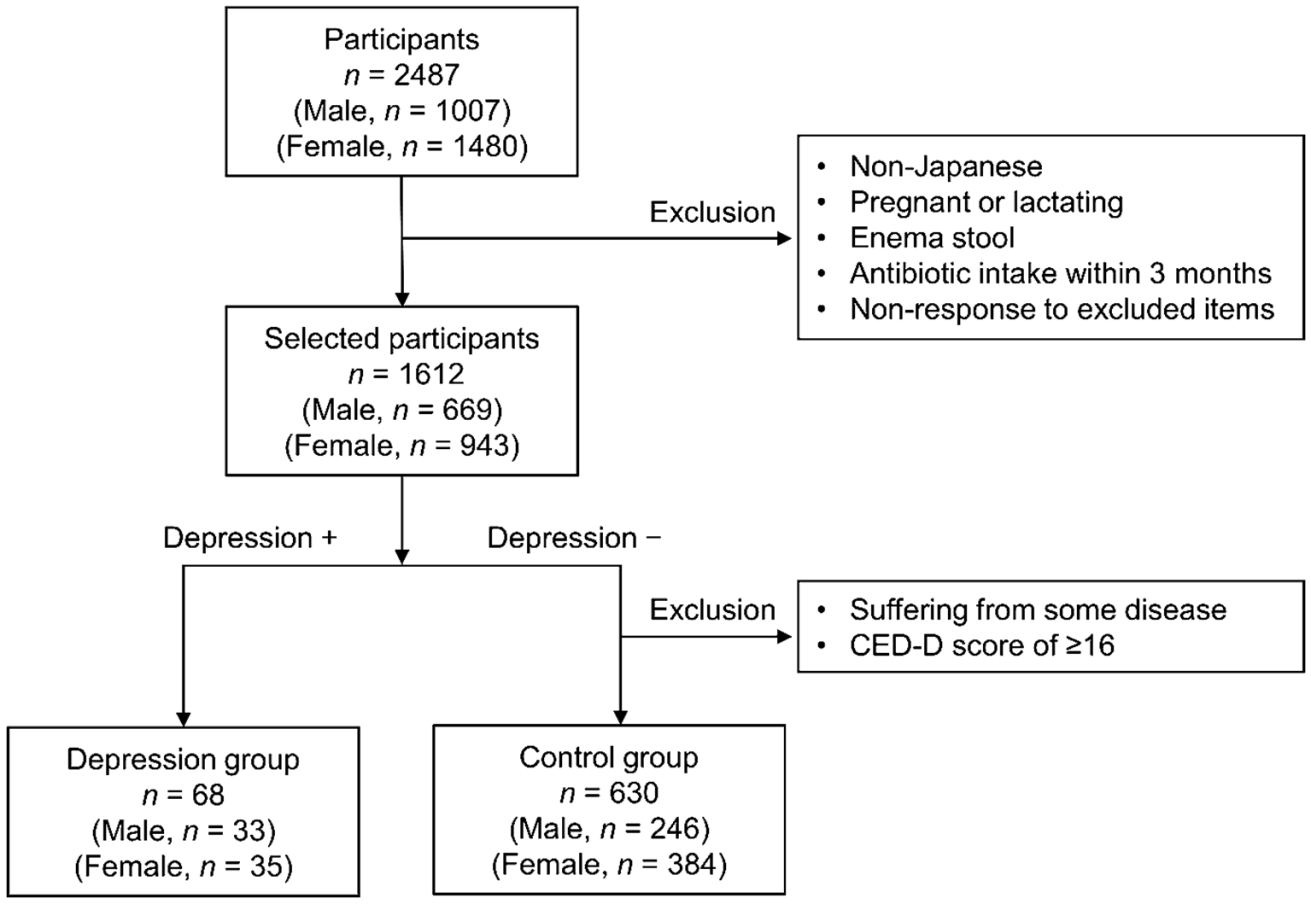
Flow chart illustrating participant screening.

The screening process implemented for study participants is shown in **Figure 1**. The exclusion criteria for this study were non-Japanese individuals, pregnant or lactating females, enema stool, antibiotic intake within 3 months, and non-response to the exclusion criteria questionnaire. Of the 1,612 participants who were not excluded, 68 (33 males ages 20 to 68 years old and 35 females ages 21 to 61 years old) with depression were included in the depression group. Among the participants who did not have depression, 630 (246 males ages 20 to 69 years old and 384 females ages 20 to 68 years old) without any current illness or treatment and with a CES-D score of < 16 [cutoff value of ≥ 16, with higher scores indicating suspicion of depression (15)] were selected as the control group.

### 2.2 Analyzing the intestinal microbiota

Stool sample collection, DNA extraction, and 16S rRNA gene sequence (variable regions V1 to V3) analysis using the MiSeq system (Illumina, San Diego, CA, USA) followed the method described by Hatayama et al. (14). Stool samples were self-collected by participants using a stool collection kit (Techno-Suruga Laboratory Co., Ltd., Shizuoka, Japan) and were mailed to the laboratory without freezing or refrigeration. The partial 16S rRNA gene sequence (variable regions V1 to V3) was determined using the 35F and 520R primers (16, 17). Amplicon sequence variants (ASVs) were created using the DADA2 v. 1.16.0 package (18) in R software v. 4.0.3 (R Foundation for Statistical Computing, Vienna, Austria) (19). The taxonomic affiliation of ASVs was determined using the Ribosomal Database Project (RDP) training set v. 18 (20) (available online at https://zenodo.org/record/4310151#.ZDUBAXbP2Ht; accessed 11 April 2023). The genus-level ASV table was rarefied based on the sequence coverage (21) (slope: 0.002259329 or less) using vegan v. 2.5.7 package (22) in R. The α-diversity analyses and comparison of intestinal microbiota between groups using the ANOVA-like differential expression tool (ALDEx2) (23–25) were conducted according to Hatayama et al. (14). The centered log ratio (CLR)-transformed intestinal microbiota data were used for the ALDEx2 comparison. To visualize β-diversity, non-metric multidimensional scaling (NMDS) based on the Bray–Curtis index was used. The metaMDS function in R v. 4.2.0 vegan v. 2.6-4 package (26) was used for NMDS. Permutational Multivariate Analysis of Variance (PERMANOVA) was performed using the vegan v. 2.6-4 package adonis function, with 9999 permutations. Permutational Multivariate Analysis of Dispersion (PERMDISP) (multivariate homogeneity of group dispersions) (27) was performed using the betadisper function in the vegan v. 2.6-4 package.

### 2.3 Statistical analysis

R (v. 4.1.0) was used for statistical analysis. Welch’s t-test and Wilcoxon rank-sum test were used to compare the data between groups. Correction for the Wilcoxon rank-sum test was performed using the Benjamini–Hochberg method. Statistical significance was set at *p*-value < 0.05.

### 2.4 Estimating depression risk

A depression risk–estimation model using SEM was constructed according to the method described by Tokuno et al. (13). The details of its construction are described by Hatayama et al. (14). The SEM was constructed using the CLR-transformed intestinal microbiota by ALDEx2 and depression incidence data. The SEMs were performed using the cfa function in the lavaan v 0.6-12 package (28). The cfa function was estimated using the diagonally weighted least-squares method. The latent variable values in the SEMs were obtained using the lavPredict function in lavaan using the Empirical Bayes method (EBM). The measurement equation portions of the SEMs were extracted to construct new SEMs used to calculate the latent variable values in the depression morbidity data–blinded situation. The latent variable values from the new SEMs were obtained using the lavPredict function with the EBM. The values of these latent variables obtained from the new SEMs were used as explanatory variables in constructing the depression risk–estimation model. The depression risk–estimation models were constructed using logistic regression analysis. Model training and 10-fold cross-validations were performed using the trainControl and train functions in the caret v 6.0-93 package (29). The themis package (v. 1.0.0) (30) was used for SMOTE sampling in the training function. The receiver operating characteristics (ROC) were analyzed and the ROC curve was drawn using the roc and ggroc functions of pROC v. 1.18.0 (31).

## 3 Results

### 3.1 Demographic characteristics

The depression and control groups were screened as shown in **Figure 1**. The differences in age or body mass index (BMI) between the depression and control groups were not significant for both sexes (**Table 1**). The CES-D scores in the depression group for both sexes were significantly higher than those in the control group (**Table 1**).

**Table 1:** Age, body mass index (BMI), and the Center for Epidemiologic Studies Depression Scale (CES-D) score of males and females in the depression and control groups.

	Depression	Control	*p*-value
Male			
Number of samples	33	246	–
Age (years)	41.5 ± 12.0	42.7 ± 11.0	0.594
BMI (kg/m^2^)	24.1 ± 4.4	23.6 ± 3.1	0.576
CES-D score	25.1 ± 13.1	6.2 ± 4.5	<0.001
Female			
Number of samples	35	384	–
Age (years)	41.3 ± 10.8	41.2 ± 10.6	0.920
BMI (kg/m^2^)	22.2 ± 4.0	20.9 ± 2.5	0.111
CES-D score	24.4 ± 12.8	6.5 ± 4.3	<0.001

Age, BMI, and CES-D show mean ± standard deviation. *p*-values of age and BMI obtained from Welch’s t-test. *p*-values of CES-D obtained from Wilcoxon rank-sum test -, not applicable.

### 3.2 Comparing intestinal microbiota between the depression and control groups

Amplicon sequencing data of the 16S rRNA gene (V1 to V3 regions) were used for diversity analysis of the intestinal microbiota. The number of reads after quality filtering and removal of chimeras ranged from 4,658 to 190,117, with an average of 40,714 reads (SD = 34,155) in 698 samples of depression and control groups. The number of reads after rarefaction ranged from 2,283 to 34,368, with an average of 9,151 reads (SD = 4,894). Overall, three indices, Shannon, Simpson, and Pielou at the genus level, were used in the α-diversity analysis. The Shannon and Simpson indices reflect community richness and evenness, while the Pielou index reflects evenness. The difference in α-diversity (the three indices) between the depression and control groups for both sexes was insignificant (**Supplementary Figure S1**). We analyzed differences in β-diversity between the depression and control groups using permutational multivariate analyses (PERMANOVA and PERMDISP). The β-diversity analysis indicated that the composition of the intestinal microbiota did not differ between the depression and control groups for males (PERMANOVA: *p* = 0.368, PERMDISP: *p* = 0.768). For females, both PERMANOVA and PERMDISP *p*-values were below 0.05 (PERMANOVA: *p*=0.005、PERMDISP: *p*<0.001). A significant result from PERMDISP would indicate that the groups differ in dispersion. Although β-diversity was visualized using a non-metric multidimensional scaling method (NMDS) derived from the Bray-Curtis index (**Supplementary Figure S2**), it exhibited high stress values (stress = 0.25) and could not be interpreted. The difference in β-diversity between the female depression and control groups could not be determined from these results.

**Figure 2:**
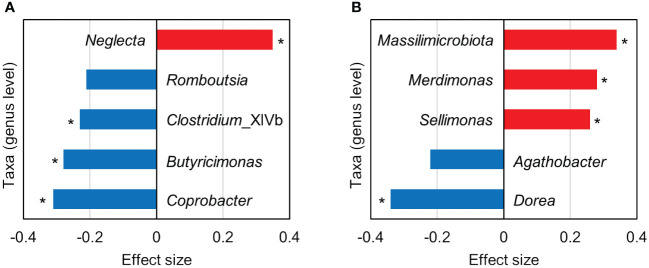
Bacterial taxa (genus level) with an absolute effect size > 0.2 based on the ALDEx2 analysis comparing the intestinal microbiota between the depression and control groups. **(A, B)** show the results for males and females, respectively. Positive (red bars) and negative (blue bars) effect sizes indicate taxa with more and less abundance, respectively, in the depression group compared with the control group. Asterisks indicate that the *p*-value of the Wilcoxon rank-sum test was < 0.05; however, the Benjamini–Hochberg corrected *p*-value was > 0.05.

Although the lack of significant difference in the overall intestinal bacterial community structure between the depression and control groups, we next evaluated the association between the specific intestinal bacteria and depression. ALDEx2 was used to identify the different intestinal bacteria in the depression and control groups. Bacterial taxa (genus level) with effect sizes > 0.2 and < −0.2 based on the ALDEx2 analysis were defined as more abundant and less abundant, respectively, in the depression group compared with the control group. In males, *Neglecta* was a more abundant taxon, and *Coprobacter*, *Butyricimonas*, *Clostridium*_XlVb, and *Romboutsia* were less abundant (**Figure 2A**). Among females, *Massilimicrobiota*, *Merdimonas*, and *Sellimonas* were observed as more abundant, whereas *Dorea* and *Agathobacter* as less abundant (**Figure 2B**).

### 3.3 Estimating depression risk using intestinal microbiota

Tokuno et al. reported a method for estimating disease risk from the intestinal microbiota composition using SEM, in which the characteristic intestinal bacteria in the disease group were used as the observational variables (13). This study attempted to create models to separately estimate depression risk in males and females using this method.

**Figure 3:**
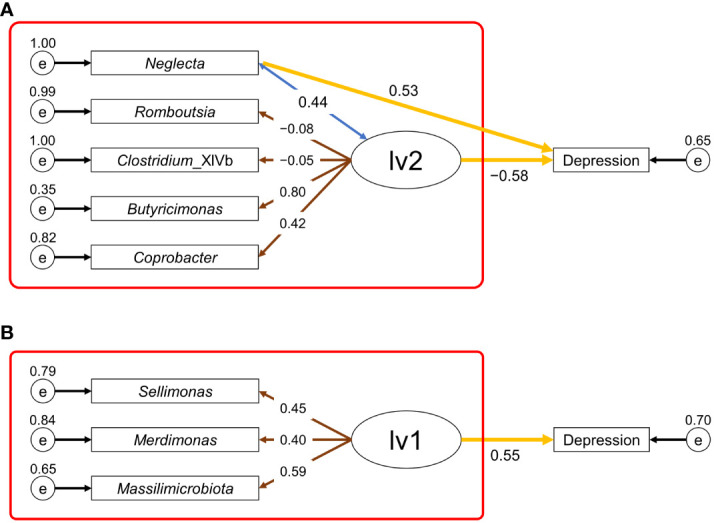
Structural equation modeling (SEM) used for depression risk estimation. **(A)**, SEM for males; **(B)**, SEM for females. Ellipses (lv1 or lv2) represent the latent variables, rectangles (depression or taxon name) represent the observed variables, and small circles (e) represent the residual terms. The values of residual variance, correlation coefficient, loading value, and path coefficient are shown near the small circle, double-headed blue arrow, brown arrow, and yellow arrow, respectively. The values of other parameters of the SEM for males **(A)** and females **(B)** are shown in **Supplementary Tables S1**, **S2**, respectively. The red box indicates the part of the SEM used for estimating the latent variable values.

First, we attempted to construct an SEM with two latent variables using data on the CLR-transformed relative abundance of intestinal bacteria as the observed variable. The latent variables 1 (lv1) and 2 (lv2) were assumed to have positive and negative effects on depression, respectively. The more and less abundant taxa in the depression group were assigned to lv1 and lv2, respectively, as the observed variables (indicators). However, for males, the only variable observed in lv1 was *Neglecta*, which was changed to a direct observation of the association with depression. Starting from the first constructed SEM, we constructed a new SEM with the observed variables reduced to meet the following conditions: goodness-of-fit index (GFI) and adjusted GFI (AGFI) close to 1, root mean square error of approximation (RMSEA) close to 0, and the absolute value of the path coefficient from the latent variable to depression incidence being the largest. **Figure 3** shows the final established SEMs. In the established SEMs, five and three intestinal bacterial taxa were assigned as the observed variables for males and females, respectively (lv2 was reduced for females). For males, an SEM was constructed with GFI = 0.92, AGFI = 0.80, RMSEA = 0.07, a path coefficient of 0.53 (*p <*0.01) from *Neglecta* to the depressed affected variable, and −0.58 (*p <*0.01) from lv2 to the depressed affected variable (**Figure 3A**). For females, an SEM was constructed with GFI = 1.00, AGFI = 1.00, RMSEA <0.01, and a path coefficient of 0.55 (*p <*0.01) from lv1 to the depression incidence variable (**Figure 3B**). Subsequently, for each sex, we constructed a new SEM based on the measurement equation portion of the established SEM in **Figure 3** (red box) to estimate the latent variable values (males: lv1; females: lv2) when the morbidity of depression in the participant was unknown. Using these SEMs, estimates of each participant’s latent variables were calculated. Finally, we constructed risk-estimation models for depression in males and females using a logistic regression model with the estimated values of the latent variables as the explanatory variables.

**Figure 4:**
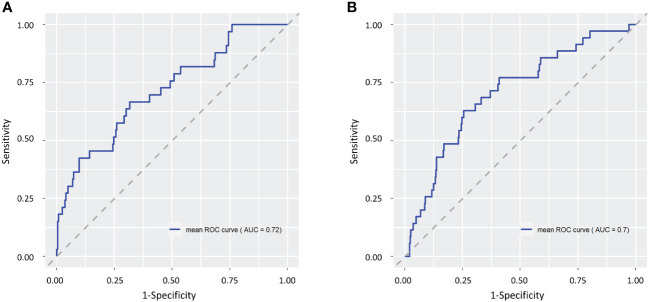
Receiver operating characteristic (ROC) curves for male **(A)** and female **(B)** depression risk-estimation models. The ROC curve for a random estimate is shown as a gray dashed line.

ROC curve analysis was performed to test the predictive accuracy of each risk-estimation model for depression (**Figure 4**). The area under the curve (AUC) values for the risk-estimation model for depression were 0.72 for males and 0.70 for females. This indicates that intestinal microbiota–based risk-estimation models for depression can discriminate between individuals with and without depression in both sexes.

## 4 Discussion

This study compared the intestinal microbiota of Japanese patients with depression with that of a control group without depression, with groups separated by sex, to investigate the intestinal microbiota associated with depression. Based on the effect size values from ALDEx2 analysis, we identified taxa of intestinal bacteria associated with depression. Among the intestinal bacteria associated with depression, *Coprobacter*, *Dorea*, and *Sellimonas* were also reported to be associated with depression in previous studies (9, 10, 32–34). *Coprobacter* and *Dorea* were reported to be present at a relatively low abundance in patients with depression (9, 32–34), whereas *Sellimonas* was present at a high abundance (10), which was consistent with the results observed in the present study. However, many of the intestinal bacteria associated with depression were not consistent between previous studies and the results of this study. One reason for this may be the fact that the previous studies were conducted on mixed-sex populations, whereas this study was conducted on separate sex groups. In fact, this study found that different taxa of intestinal bacteria were associated with depression in male and female populations. Therefore, the lack of consensus in previous studies on the taxa of intestinal bacteria associated with depression may be due to the sex composition of the study populations. In addition, intestinal microbiota composition is also related to ethnicity, geographic location, and other factors (35). Different study populations may have influenced the differences in the results of previous studies.

Based on published papers, we looked at known metabolic products of intestinal bacteria that were associated with depression. Taxa with more abundance in the depression group than in the control group included *Neglecta* in males and *Massilimicrobiota*, *Merdimonas*, and *Sellimonas* in females. Acetic acid is the main metabolic end product of *Merdimonas* and *Sellimonas* (36, 37). However, the metabolic products of *Neglecta* and *Massilimicrobiota* are unknown. *Coprobacter*, *Butyricimonas*, *Clostridium*_XlVb, and *Romboutsia* in males and *Dorea* and *Agathobacter* in females were the taxa with less abundance in the depression group. *Coprobacter* produces propionic and acetic acids as its major final metabolic products and a small amount of succinic acid (38). *Butyricimonas* produces hydrogen (H_2_) and butyric acid as the main final metabolic products and acetic acid, propionic acid, succinic acid, and isobutyric acid to a lesser extent (39). *Clostridium colinum* belonging to *Clostridium*_XlVb produces acetic acid, ethanol, and short-chain fatty acids (40). *Romboutsia* produces acetic acid and formic acid as its main final metabolic products and also produces H_2_ (41). *Dorea* produces H_2_, and acetic acid, formic acid, and ethanol as its main metabolic end products (42). Lactic acid may or may not be formed. *Agathobacter* produces H_2_, and its main end products of metabolism are butyric, acetic, and lactic acids (43). A previous study reported that intestinal bacteria associated with depression are involved in the synthesis of glutamate, serotonin, and GABA, which are key neurotransmitters for depression (10). However, it was unclear whether the taxa of intestinal bacteria shown to be associated with depression in this study are related to these metabolites.

A previous study in a Japanese population reported that the intestinal microbiota may influence the depressive state of the host via butyrate-producing process (9). Butyric acid-producing bacteria (*Butyricimonas* in males and *Agathobacter* in females) were also observed in this study as intestinal bacteria associated with depression. However, we could not find any previous reports on *Neglecta* and *Massilimicrobiota* (more abundant taxa in the depression group) that contained information related to their metabolic products. Therefore, determining the effects of metabolic products including butyric acid on depression was challenging. Nevertheless, it is interesting to note that H_2_ producing capacity was observed in intestinal bacteria that were less abundant in the depression group of both sexes (males: *Butyricimonas* and *Romboutsia*; females: *Dorea* and *Agathobacter*). H_2_ could play an important role in alleviating oxidative stress and reducing inflammation *in vivo* (44). H_2_ is also capable of entering and exiting the bloodstream, diffusing, passing through selection barriers such as the blood–brain barrier, and acting at various sites, including the brain. The exacerbation of brain inflammation has been suggested to be associated with depression (45). Furthermore, H_2_ gas inhalation reduces oxidative stress and suppresses symptoms in mice with mild traumatic brain injury that exhibit depression-like behaviors (46). Considering these facts, the intestinal microbiota in patients with depression may produce lesser H_2_, reducing brain H_2_ levels, thus affecting functions that suppress the depression-associated inflammation in the brain.

The taxon names, *Neglecta* and *Massilimicrobiota*, are based on the RDP database [training set v. 18 (20)]; however, they are not validly published names (47) (https://lpsn.dsmz.de/ accessed on February 1, 2024). *Neglecta* has been proposed to be classified to *Neglectibacter* (48); however, *Neglectibacter* is also not recognized as a valid name. There is no information on these taxa such as their metabolic products, and further research to identify these products is desirable to understand their association with depression. Further studies are needed to determine why these intestinal bacteria are more abundant in patients with depression and how they affect them.

The intestinal bacteria associated with depression differed according to sex. This may be owing to the influence of sex differences on intestinal microbiota. However, a reduction in the prevalence of H_2_ producing bacteria was observed in the depression group of both sexes. Although intestinal bacteria associated with depression may be sex-specific in terms of taxa, they may play a similar functional role.

Herein, the α-diversity of the intestinal microbiota was not significantly different between the depression and control groups, which was similar to the results of previous studies (33, 34, 49, 50). Varying results regarding the significant differences in β-diversity have been reported in various studies (33, 34, 50). In terms of the diversity of the intestinal microbiota, distinguishing between the depression and control groups may be challenging.

The risk-estimation models for depression constructed in this study were able to discriminate individuals with depression from those without depression (AUC: 0.72 for males and 0.70 for females). In general, an AUC value of 0.5 suggests no discrimination, 0.7–0.8 is acceptable, 0.8–0.9 is excellent, and higher than 0.9 is considered outstanding (51). Briefly, the accuracy of this risk estimation models was moderate. The method of estimating the depression risk using these models can be incorporated into the testing of the intestinal microbiota and is expected to be useful for efficient screening of individuals with depression and as an aid in diagnosing depression. Early detection and treatment of individuals at high risk of depression and those suffering from depression can prevent depression-related suicides and reduce the risk of recurrence. Furthermore, it would lighten the burden on the healthcare system.

Currently, depression is primarily treated using psychotherapy and pharmacotherapy. Pharmacotherapy uses antidepressants such as selective serotonin reuptake inhibitors (SSRIs). Antidepressants are associated with a small number of side effects, including nausea, dry mouth, headache, diarrhea, tremor, dizziness, anxiety, nervousness, agitation, insomnia, constipation, and sexual side effects (4, 52). Therefore, even if the medication alleviates the symptoms of depression, the patient is at risk of suffering from unpleasant side effects. There is also concern about the so-called discontinuation syndrome or withdrawal syndrome when SSRIs are abruptly or gradually discontinued (4, 53, 54). Depression-associated intestinal bacteria identified in this study may serve as the intervention targets for diet and dietary supplementation. These findings could lead to the development of strategic food-based interventions for depression as an alternative to pharmacotherapy.

This study had some limitations. First, the sample size, particularly of the depression group, used in this study was small, which affects the statistical significance of the results. Therefore, we focused on the effect sizes independent of the sample size and identified the intestinal bacteria that demonstrated differences between the depression and control groups. Second, information on depression, including type of depression, psychiatric medication use, childhood trauma, and quality of life scores, was not collected and was not considered in the study. Third, this study failed to control for confounding factors related to diet and living environment. Fourth, considering that the intestinal microbiota composition is associated with ethnicity and geographic location (35), the results of this study may be limited to the Japanese population. To understand the association between depression and intestinal microbiota in more detail, future studies with larger sample sizes that account for more confounding factors are needed. Furthermore, risk-estimation models for depression based on the results could further improve their accuracy.

## 5 Conclusions

This study demonstrated that depression is associated with the intestinal microbiota. Although the taxa of intestinal bacteria associated with depression differed according to sex, dysbiosis, which is characterized by fewer H_2_-producing bacteria, was observed in the depression group. These results led to a new hypothesis that patients with depression have a reduced amount of H_2_ derived from the intestinal bacteria, which performs an anti-inflammatory function in the brain and are, therefore, unable to suppress inflammation in the brain due to intense stress. We envision that future studies will test this hypothesis, leading to a better understanding of the mechanisms underlying the onset and progression of depression and the development of treatment and prevention methods.

The incorporation of this method of estimating the depression risk in stool sample–based testing of the intestinal microbiota could be an effective screening tool for individuals suffering from depression or at high risk for depression. Additionally, the results of the depression risk estimation are expected to provide useful information for diagnosing depression. Targeting the intestinal bacteria associated with depression identified in this study may lead to the development of strategic food-based interventions. Thus, the findings of this study are expected to be useful for early detection and treatment of individuals with depression.

## Data availability statement

The datasets presented in this study are not publicly available because of privacy concerns. Requests to access the data should be directed to the corresponding author.

## Ethics statement

The studies involving humans were approved by The Ethics Committee of Shiba Palace Clinic (Tokyo, Japan). The studies were conducted in accordance with the local legislation and institutional requirements. The participants provided their written informed consent to participate in this study.

## Author contributions

KO: Writing – original draft, Visualization, Methodology, Investigation, Formal analysis, Data curation. KH: Writing – review & editing, Visualization. HT: Writing – review & editing, Formal analysis, Data curation. AE: Writing – review & editing, Investigation, Formal analysis, Data curation. AO: Writing – review & editing, Investigation. HM: Writing – review & editing, Supervision, Resources, Project administration, Funding acquisition, Conceptualization. NH: Writing – review & editing, Resources. NT: Writing – review & editing, Resources.
