# Supplementary material for: A risk estimation method for depression based on the dysbiosis of intestinal microbiota in Japanese patients
Source: Front Psychiatry. 2024 May 28;15:1382175. doi: 10.3389/fpsyt.2024.1382175 (PMC11165696; doi:10.3389/fpsyt.2024.1382175)
Supplement: Supplementary file 1 [file DataSheet_1.pdf]

Supplementary Material

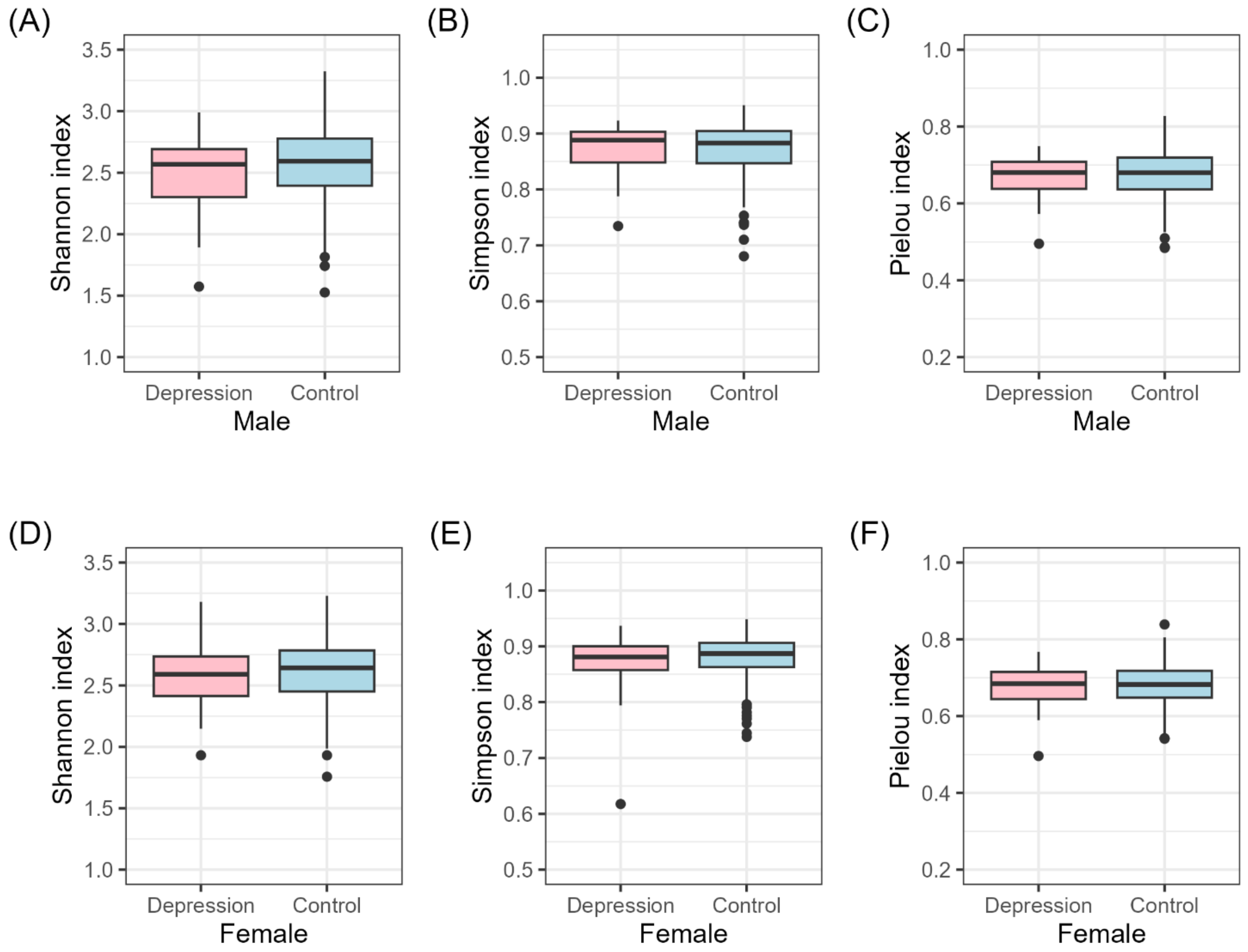

Supplementary Figure S1. α-diversity analysis of the intestinal microbiota in the depression and control groups. α-diversity indices of the Shannon (A, D), Simpson (B, E), and Pielou (C, F) indices are shown. Panels (A) – (C) and (D) – (F) show the results of the male and female subgroups, respectively. The difference in α-diversity (Shannon, Simpson, and Pielou indices) between the depression and control groups for both sexes was insignificant (Welch's t-test, statistically significant difference: $p < 0.05$).

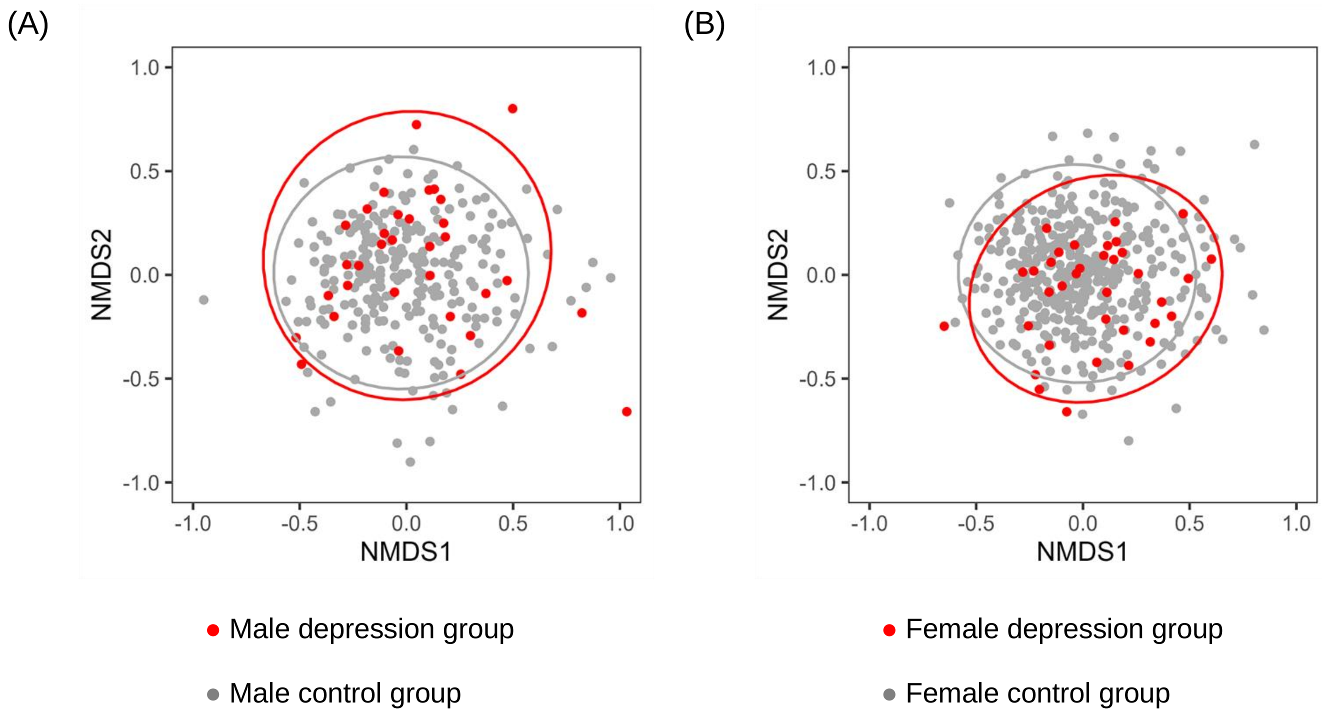

Supplementary Figure S2. Non-metric multidimensional scaling (NMDS) plot of the intestinal microbiota for males (A) (stress = 0.23) and females (B) (stress = 0.25). Stress >0.2 indicates that differences should be interpreted with caution. Samples from the depression and control groups are indicated with red and gray dots, respectively, in the NMDS plots. Around the centroid, 95% confidence intervals are indicated as ellipses.

Supplementary Table S1. The parameters for male structural equation modeling shown in Figure 3a. NA, not available.

lhs[a]	op[b]	rhs[c]	est.std[d]	se[e]	z[f]	*p*-value	ci.lower[g]	ci.upper[h]
lv2	=~	*Coprobacter*	0.42	0.07	5.93	0.00	0.28	0.56
lv2	=~	*Butyricimonas*	0.80	0.11	7.50	0.00	0.59	1.01
lv2	=~	*Clostridium*_XlVb	−0.05	0.07	−0.74	0.46	−0.20	0.09
lv2	=~	*Romboutsia*	−0.08	0.07	−1.04	0.30	−0.22	0.07
Depression	~	*Neglecta*	0.53	0.13	4.04	0.00	0.27	0.79
Depression	~	lv2	−0.58	0.15	−3.84	0.00	−0.87	−0.28
lv2	~~	*Neglecta*	0.44	0.08	5.51	0.00	0.28	0.59
Coprobacter	~~	*Coprobacter*	0.82	0.06	13.59	0.00	0.70	0.94
Butyricimonas	~~	*Butyricimonas*	0.35	0.17	2.05	0.04	0.02	0.69
*Clostridium*_XlVb	~~	*Clostridium*_XlVb	1.00	0.01	123.55	0.00	0.98	1.01
Romboutsia	~~	*Romboutsia*	0.99	0.01	90.86	0.00	0.97	1.02
Depression	~~	*Depression*	0.65	0.15	4.48	0.00	0.37	0.94
Neglecta	~~	*Neglecta*	1.00	0.00	NA	NA	1.00	1.00
lv2	~~	lv2	1.00	0.00	NA	NA	1.00	1.00
Coprobacter	~1		0.00	0.06	0.00	1.00	−0.12	0.12
Butyricimonas	~1		0.00	0.06	0.00	1.00	−0.12	0.12
*Clostridium*_XlVb	~1		0.00	0.13	0.00	1.00	−0.26	0.26
Romboutsia	~1		0.00	0.06	0.00	1.00	−0.12	0.12
Depression	~1		0.00	0.00	NA	NA	0.00	0.00
Neglecta	~1		0.00	0.07	0.00	1.00	−0.15	0.15
lv2	~1		0.00	0.00	NA	NA	0.00	0.00

[a] Variable name that appears on the formula's left side
[b] Operator indicates the following formula types: =~, latent variable definition; ~, regression; ~~, (residual) (co)variance; and ~1, intercept
[c] Variable name that appears on the formula's right side
[d] Standardized parameter estimates
[e] Standard error of the estimated parameter
[f] Z-value
[g] Lower 95% confidence intervals
[h] Upper 95% confidence intervals

Supplementary Table S2. The parameters for the female structural equation modeling shown in Figure 3b. NA, not available.

lhs[a]	op[b]	rhs[c]	est.std[d]	se[e]	z[f]	*p*-value	ci.lower[g]	ci.upper[h]
lv1	=~	*Massilimicrobiota*	0.59	0.08	7.64	0.00	0.44	0.74
lv1	=~	*Merdimonas*	0.40	0.07	5.53	0.00	0.26	0.55
lv1	=~	*Sellimonas*	0.45	0.07	6.42	0.00	0.32	0.59
Depression	~	lv1	0.55	0.15	3.70	0.00	0.26	0.84
Massilimicrobiota	~~	*Massilimicrobiota*	0.65	0.09	7.06	0.00	0.47	0.83
Merdimonas	~~	*Merdimonas*	0.84	0.06	14.20	0.00	0.72	0.95
Sellimonas	~~	*Sellimonas*	0.79	0.06	12.39	0.00	0.67	0.92
Depression	~~	Depression	0.70	0.16	4.26	0.00	0.38	1.02
lv1	~~	lv1	1.00	0.00	NA	NA	1.00	1.00
Massilimicrobiota	~1		0.00	0.05	0.00	1.00	−0.10	0.10
Merdimonas	~1		0.00	0.09	0.00	1.00	−0.19	0.19
Sellimonas	~1		0.00	0.05	0.00	1.00	−0.10	0.10
Depression	~1		0.00	0.00	NA	NA	0.00	0.00
lv1	~1		0.00	0.00	NA	NA	0.00	0.00

[a] Variable name that appears on the formula's left side
[b] Operator indicates the following formula types: =~, latent variable definition; ~, regression; ~~, (residual) (co)variance; and ~1, intercept
[c] Variable name that appears on the formula's right side
[d] Standardized parameter estimates
[e] Standard error of the estimated parameter
[f] Z-value
[g] Lower 95% confidence intervals
[h] Upper 95% confidence intervals
